# Quantitative electroencephalography parameters as neurophysiological biomarkers of schizophrenia-related deficits: A Phase II substudy of patients treated with iclepertin (BI 425809)

**DOI:** 10.1038/s41398-022-02096-5

**Published:** 2022-08-11

**Authors:** Christian Schultheis, Holger Rosenbrock, Salome Rebecca Mack, Richard Vinisko, Niklas Schuelert, Andrea Plano, Sigurd D. Süssmuth

**Affiliations:** 1 Boehringer Ingelheim Pharma GmbH & Co. KG, Biberach an der Riss, Germany; 2 Boehringer Ingelheim Pharmaceuticals Inc., Ridgefield, CT USA; 3 Boehringer Ingelheim International GmbH, Biberach an der Riss, Germany

**Keywords:** Diagnostic markers, Schizophrenia

## Abstract

Patients with schizophrenia experience cognitive impairment related to neural network dysfunction and deficits in sensory processing. These deficits are thought to be caused by *N*-methyl-D-aspartate receptor hypofunction and can be assessed in patient populations using electroencephalography (EEG). This substudy from a Phase II, randomized, double-blind, placebo-controlled, parallel-group study investigating the safety and efficacy of the novel glycine transporter-1 inhibitor, iclepertin (BI 425809), assessed the potential of EEG parameters as clinically relevant biomarkers of schizophrenia and response to iclepertin treatment. Eligible patients were randomized to once-daily add-on iclepertin (2, 5, 10, or 25 mg), or placebo (1:1:1:1:2 ratio) for 12 weeks. EEG data were recorded from a subgroup of patients (*n* = 79) at baseline and end of treatment (EoT). EEG parameters of interest were mismatch negativity (MMN), auditory steady-state response (ASSR), and resting state gamma power, and their correlations with clinical assessments. At baseline, MMN and ASSR exhibited consistent correlations with clinical assessments, indicating their potential value as neurophysiological biomarkers of schizophrenia-related deficits. ASSR measures were positively correlated to the MATRICS Consensus Cognitive Battery overall and neurocognitive composite scores; MMN amplitude was positively correlated with Positive and Negative Syndrome Scale scores. However, correlations between change from baseline (CfB) at EoT in clinical assessments, and baseline or CfB at EoT for EEG parameters were modest and inconsistent between dose groups, which might indicate low potential of these EEG parameters as predictive and treatment response biomarkers. Further methodological refinement is needed to establish EEG parameters as useful drug development tools for schizophrenia.

## Introduction

Schizophrenia is a complex neuropsychiatric disorder characterized by positive, negative, and cognitive symptoms [1, 2]. In particular, cognitive impairment associated with schizophrenia (CIAS) represents an area of great unmet medical need due to the lack of effective pharmacotherapies specifically targeting these symptoms [3]. CIAS and other symptoms are related to persistent neurocognitive deficits in sensory processing and neural network function, which can be assessed in patients with schizophrenia using electroencephalography (EEG) [4–6]. Since many EEG parameters relevant to CIAS are phylogenetically conserved, they have considerable potential as translatable biomarkers for the development of novel pharmacotherapies [4, 7]. However, further validation is required to ensure the reproducibility of these measures and to establish their use in a real-world clinical setting.

One group of EEG measures with potential as translatable biomarkers are auditory event-related potentials (AERPs). Patients with schizophrenia exhibit deficits in AERPs, such as mismatch negativity (MMN) [4–7]. MMN is a pre-attentive sensory response to deviating auditory stimuli, which occurs approximately 50 ms after the onset of a deviant stimulus, and peaks approximately 100–150 ms later [8]. It is typically elicited in response to an auditory oddball paradigm, whereby a pattern of repeated stimuli is interrupted by a physically deviant stimulus, such as simple changes in frequency and duration or violations of complex patterns or abstract rules [9]. The MMN is a negative component of a waveform, derived from the difference wave subtracting the response to the standard from the response to the deviant signal. In patients with schizophrenia, MMN amplitude is commonly reduced, resulting in less negative MMN amplitudes compared with healthy individuals [10]. Sensory processing dysfunction is believed to contribute significantly to MMN deficits observed among patients [9].

Patients with schizophrenia also display deficits in power and phase-locking of 40 Hz auditory steady-state response (ASSR), a measure of the capacity of auditory circuits to entrain to a frequency-modulated stimulus [4–6]. Measurements of ASSR probe the integrity of sensory pathways and the ability to synchronize and maintain precisely coordinated activity. Additionally, patients exhibit bidirectional disturbances in resting-state local field potential oscillations in the gamma frequency range (~25–100 Hz), which are thought to enable coordinated network activity during normal brain functioning and are integral to several aspects of learning and memory [11–13].

It is likely that these deficits are directly related to *N*-methyl-D-aspartate receptor (NMDAR) hypofunction and impaired glutamatergic signaling [12, 14–16], which are implicated in the pathophysiology of CIAS [17, 18]. Under non-pathological conditions, NMDARs play a key role in learning and memory by mediating neural synchrony [11] and synaptic plasticity [19, 20]. However, in patients with schizophrenia, NMDAR hypofunction is believed to underlie reduced functional inhibition by interneurons and the subsequent disinhibition of pyramidal cells, leading to an excitatory/inhibitory (E/I) imbalance and perturbed network function in prefrontal cortex [12, 14–16]. The resulting disruption of sensory processing and neural synchrony [12, 14, 15] underlies sensory and neural network deficits in patients with schizophrenia, which are reflected by changes in AERPs, such as MMN and ASSR [4, 5, 7, 21, 22].

Thus, targeting NMDAR hypofunction to enhance glutamatergic signaling offers a promising therapeutic strategy to ameliorate sensory processing deficits and effectively treat CIAS [19]. Indeed, data from clinical trials indicates that schizophrenia-related deficits in MMN and ASSR can be attenuated by agents targeting NMDA-receptor signaling, such as glycine [23] and D-serine [24], coinciding with improvements in clinical symptoms associated with schizophrenia. However, in a small clinical study, the GlyT1 inhibitor, bitopertin, had no significant effect on MMN at the dose tested [25], and after a positive Phase II study [26], it later failed in Phase III of its clinical development due to the lack of improvement in negative symptoms over placebo [27]. In addition to highlighting the potential benefits of NMDAR modulation for the treatment of schizophrenia, these findings also support the further investigation of EEG parameters as biomarkers for NMDAR hypofunction in patients, and as biomarkers of treatment response with agents targeting NMDAR or glutamatergic signaling.

Iclepertin (BI 425809) is a novel glycine transporter-1 (GlyT1) inhibitor currently under development for the treatment of CIAS [28–31]. Treatment with iclepertin is thought to enhance NMDAR function and glutamatergic signaling by increasing the synaptic concentration of glycine, an obligatory co-agonist at NMDARs [28–31]. Phase I and II clinical trials have demonstrated that iclepertin is a potent and selective inhibitor of GlyT1 that indirectly shows central target engagement by increasing glycine levels in the cerebrospinal fluid of healthy volunteers [30], is well tolerated in healthy volunteers at doses up to 75 mg [28–30], and has shown pro-cognitive effects in a Phase II study in patients with schizophrenia [31]. In particular, treatment with iclepertin 10 and 25 mg led to greater improvements from baseline in the MATRICS Consensus Cognitive Battery (MCCB) overall composite T-score at Week 12 versus placebo [31]. For the individual MCCB subdomain tests, the largest separation from placebo was seen for the Trail Making Test (TMT; processing speed), the Neuropsychological Assessment Battery (NAB) mazes subset (reasoning and problem solving), and the Wechsler Memory Scale, 3rd edition Spatial Span subset (WMS-III SS; working memory) [31].

As part of that 12-week Phase II trial investigating the safety and efficacy of iclepertin [31], this substudy aimed to assess the validity of EEG parameters as clinically relevant biomarkers of CIAS and other schizophrenia-related deficits, and the effects of iclepertin treatment on EEG parameters in a multicenter setting. In particular, we aimed to evaluate EEG parameters as neurophysiological biomarkers of schizophrenia-related deficits, and as biomarkers of treatment response or predictors of treatment outcomes in patients, by quantifying the correlation of EEG parameters with clinical assessments at baseline and end of treatment (EoT).

## Methods

### Eligibility criteria

Full eligibility criteria for the parent trial have been previously described [31]. In brief, male or female outpatients aged 18–50 years with schizophrenia on stable treatment with no diagnosis of any other major psychiatric disorder were included. Additional selected eligibility criteria are shown in Supplementary Table 1.

### Study design

The parent trial was a randomized, double-blind, placebo-controlled, parallel-group, Phase II study [31] (NCT02832037). Eligible patients were randomized to once-daily add-on iclepertin (BI 425809) (2, 5, 10, or 25 mg), or placebo in a 1:1:1:1:2 ratio for 12 weeks. Measurements of EEG parameters were taken from a subgroup of patients within 14 days prior to randomization (Day 1 of the parent trial), and 7 days prior to EoT (Week 12 of the parent trial) (Supplementary Fig. 1). The trial was done in accordance with the principles of the Declaration of Helsinki, the International Conference on Harmonization Guideline for Good Clinical Practice, all applicable regulatory requirements, and standard operating procedures of the sponsor. The study procedures and protocol were reviewed and approved by the independent ethics committee of the study centers and the relevant local authorities.

### Substudy endpoints

The substudy endpoints were baseline and change from baseline (CfB) at EoT in resting-state quantitative qEEG, MMN, and ASSR parameters, and their correlations with clinical assessments. These endpoints were designed to evaluate the suitability of EEG parameters as neurophysiological, treatment response, and predictive biomarkers in schizophrenia. For evaluation of EEG parameters as treatment response or predictive biomarkers, data from the iclepertin 10 mg and iclepertin 25 mg treatment groups were combined (iclepertin 10 + 25 mg), since both of these doses demonstrated improvements in MCCB scores in the parent trial [31].

### Clinical assessments

Clinical assessments of cognition and positive and negative symptoms were performed at baseline (within 14 days prior to randomization) and EoT (within 7 days prior to last dose of iclepertin at Week 12). Clinical assessments comprised: MCCB overall composite T-score, MCCB neurocognitive composite T-score, and Positive and Negative Syndrome Scale (PANSS) total score and subscales. Individual test scores for the MCCB subdomains Letter–Number Span task (LNS), WMS-III SS, NAB, and TMT Part A (TMT-A) were also assessed.

### EEG recordings

Assessment of EEG parameters occurred at baseline and EoT. Recordings were collected using an EEG cap with electrodes positioned according to the international 10–20 Jasper system and completed by nine additional electrodes (FC1/2/5/6, CP1/2/5/6, and OZ). Ear electrodes (A1 and A2) were used for mapping reference. Two electro-oculogram (EOG) channels and one electromyography (EMG) channel were recorded using silver cup electrodes to allow to improve artifact rejection. Vertical and horizontal bipolar EOG channels were used to provide information on eye/eyelid movement. The EMG electrode was placed on the middle of the nose and linked to the ears for reference.

Recordings of EEG, EOG, and EMG data were made using a Grael EEG 4 K system (sampling frequency 512 Hz; Compumedics Europe GmbH, Freiberg, Germany). Raw EEG data were recorded with a 0.1–70 Hz filter. Digital narrow notch filters centered at 50 and 60 Hz were applied to reduce the electromagnetic noise from the main power supply. Operational implementation of EEG recordings, including set-up at the study sites, and data evaluation and preparation was carried out by Biotrial S.A.S (Rennes, France). Artifact rejection, spectral analysis, and AERP constructions were performed using CURRY 8.0 (Compumedics) and Matlab (MathWorks Inc., CA, USA). Trained clinical staff recorded EEG data at each site. During recording, patients sat on an armchair bed in a quiet environment. Patients were asked to focus on a visual fixation point to minimize eye movements.

The following EEG parameters of interest were included: MMN amplitude (deviants: frequency, duration, and frequency + duration), ASSR (evoked power, induced power, and phase-locking factor [PLF]), and absolute and relative gamma power at resting state.

#### Resting state qEEG

Resting state qEEG data were recorded for 5 min in eyes-closed condition, during which the patient was asked to avoid talking, moving, sleeping, or blinking. After recording, qEEG data were visually inspected and artifacts were rejected. Data were processed using the Hanning window and Fast Fourier Transform algorithm to derive the absolute and relative power of the following frequency bands: delta (1.5–6 Hz), theta (6–8.5 Hz), alpha (8.5–12.5 Hz), beta (12.5–30 Hz), and gamma (30–40 Hz). The relative power of individual frequency bands was calculated as its absolute power divided by the absolute power of the entire spectrum from 1.5 to 30 Hz.

#### MMN

MMN was assessed in an auditory oddball task, whereby frequent standard tones (1000 Hz, 50 ms duration) were presented with 85% probability interspersed with three different infrequent deviant tones with 5% probability each: frequency deviant (1500 Hz, 50 ms), duration deviant (1000 Hz, 100 ms), or frequency + duration deviant (1500 Hz, 100 ms). All tones had 5 ms rise/fall times and were presented through earphones at 80 dB SPL, with a stimulus-onset asynchrony of 500 ms.

The EEG data were high-pass filtered (at 1 Hz), and eyeblink artifacts were removed. After baseline correction, bad epochs were detected, rejected, and interpolated as previously described [32]. Epochs with extreme values and low signal-to-noise ratio were also removed as previously described [33]. Next, a second baseline correction and a 30 Hz zero phase shift low pass filter were applied. Deviant standard difference waves were then generated by subtracting the standard from the respective deviant wave, allowing identification and peak amplitude measurements of pitch-deviant, duration-deviant, and double-deviant MMN.

MMN was estimated as the most negative peak in the time window of 100–205 ms post stimulus. This time window for detection of MMN amplitude was selected based on post hoc analyses for each deviant individually and was found most suitable for the detection of the “true” MMN peaks for all deviants, as detected by visual inspection. Further quality criteria were applied for MMN signals to be considered for inclusion in the final analyses set. For example, the baseline signal at the Fz electrode was required to remain between +2 and −2 µV, and the signal-to-noise ratio (calculated as the ratio of signal strength in the 400 ms after stimulus to the signal strength in the 100 ms preceding the signal) was required to be above 2.

#### 40 Hz ASSR

The 40 Hz ASSR paradigm assessed the gamma activity generated by repetitive auditory stimulation, which consisted of 150 click trains, each of 500 ms duration and comprising 20 clicks separated by 25 ms, presented at 80 dB through earphones. Stimulations at 20 and 30 Hz were also tested; the respective click trains were presented in a pseudo-random order across patients, while the same order was maintained within patients across baseline and end of trial assessments. During the assessment, patients were instructed to focus on a visual fixation point while listening passively to the presented auditory stimuli.

After high-pass filtering (at 1 Hz) and removal of eyeblink artifacts, 6-period Morlet wavelets were used for data processing. Next, complex time frequency representation (TFR) of each artifact-free epoch was estimated on [−1.3, +1.75] s × [4, 100] Hz domain. This set of TFRs was used for the computation of PLF and induced power. The average PLF and induced power were estimated for 100 ms segments from 100 to 500 ms in the range of 39–41 Hz. The same tools were used on the average AERP to generate a TFR that was used to estimate evoked power and the same set of basic parameters on 100 ms bins. As a quality criterion, only signals exceeding a signal to noise ratio of 4 were considered in final analysis set.

## Results

### Substudy disposition and baseline sociodemographic and clinical characteristics

A total of 79 patients from 17 sites across five countries (Germany, Italy, Poland, UK, and USA) were randomized into the EEG substudy, and 57 patients completed the substudy (Supplementary Fig. 2). The number of patients included in each analysis varied due to missing data or the exclusion of data that did not meet predefined quality control criteria, therefore n numbers for individual measurements presented here may vary. The majority of patients were White (*n* = 51; 64.6%), male (*n* = 61; 77.2%), and aged 18–40 years (*n* = 48; 60.8%), with a mean (standard deviation) age of 37.8 years (7.1) (Table 1). Baseline clinical characteristics were similar between patients included in the EEG substudy and the overall population in the parent trial [31], except for lower proportions of patients in the parent trial who were White (*n* = 237, 47.0%) or male (*n* = 329, 65.0%) [31]. There were differences in baseline cognitive impairment and symptom severity between treatment groups within the substudy, as indicated by mean MCCB overall and neurocognitive composite T-scores and PANSS total and subscale scores (Table 1).

**Table 1:** Patient demographics and baseline clinical characteristics.

	Treatment group					
	2 mg *N* = 14	5 mg *N* = 10	10 mg *N* = 20	25 mg *N* = 15	Placebo *N* = 20	Total *N* = 79
Age, years, mean (SD)	35.8 (7.9)	37.3 (6.2)	40.0 (7.1)	36.9 (7.2)	38.2 (7.1)	37.8 (7.1)
18–40 years, *n* (%)	10 (71.4)	7 (70.0)	10 (50.0)	9 (60.0)	12 (60.0)	48 (60.8)
41–50 years, *n* (%)	4 (28.6)	3 (30.0)	10 (50.0)	6 (40.0)	8 (40.0)	31 (39.2)
Male, *n* (%)	11 (78.6)	7 (70.0)	18 (90.0)	11 (73.3)	14 (70.0)	61 (77.2)
Race, *n* (%)						
Black or African American	2 (14.3)	3 (30.0)	8 (40.0)	6 (40.0)	7 (35.0)	26 (32.9)
White	11 (78.6)	7 (70.0)	11 (55.0)	9 (60.0)	13 (65.0)	51 (64.6)
Multiple race	1 (7.1)	0 (0.0)	0 (0.0)	0 (0.0)	0 (0.0)	1 (1.3)
Region, *n* (%)						
North America	8 (57.1)	5 (50.0)	11 (55.0)	7 (46.7)	8 (40.0)	39 (49.4)
Europe	6 (42.9)	5 (50.0)	9 (45.0)	8 (53.3)	12 (60.0)	40 (50.6)
Baseline clinical scores, mean (SD)						
MCCB overall composite T-score	27.5 (11.2)	37.2 (9.3)	28.1 (10.6)	27.4 (15.6)	33.3 (10.3)	30.3 (11.8)
MCCB neurocognitive T-score	29.0 (11.5)	37.5 (8.5)	30.3 (9.8)	29.1 (16.3)	33.4 (9.4)	31.5 (11.5)
PANSS total score	65.6 (16.0)	57.9 (16.1)	63.7 (19.3)	55.2 (16.3)	57.6 (19.9)	60.1 (18.0)
PANSS negative subscale	18.0 (4.7)	15.7 (5.5)	18.2 (6.4)	14.7 (4.3)	15.3 (5.0)	16.4 (5.4)
PANSS positive subscale	14.7 (6.1)	13.7 (6.2)	14.1 (5.8)	13.7 (6.0)	13.2 (6.3)	13.9 (5.9)
Concomitant antipsychotic therapy, *n* %	14 (100.0)	10 (100.0)	19 (95.0)	15 (100.0)	20 (100.0)	78 (98.7)

*MATRICS* Measurement and Treatment Research to Improve Cognition in Schizophrenia; *MCCB* MATRICS Consensus Cognitive Battery; *PANSS* Positive and Negative Syndrome Scale; *SD* standard deviation.

In the overall study, treatment with iclepertin 10 mg or 25 mg led to greater improvements from baseline in the mean (standard error [SE]) MCCB overall composite T-score at Week 12, versus placebo (3.49 [0.64] and 3.23 [0.64], versus 1.50 [0.46]) [31]. Similar findings were observed for the EEG substudy population, with a mean (SE) change from baseline in MCCB overall composite score in the combined iclepertin 10 + 25 mg group of 4.25 (1.12), versus −0.09 (1.41) for placebo.

### EEG parameters as neurophysiological biomarkers associated with schizophrenia-related deficits at baseline

**Fig. 1:**
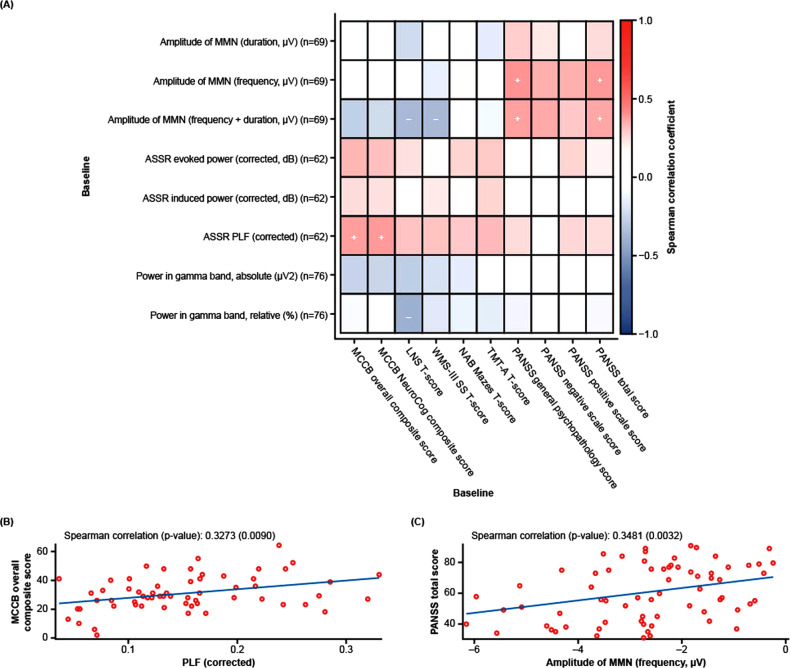
EEG parameters as neurophysiological biomarkers. Spearman correlation of amplitude of MMN deviants, ASSR parameters, and gamma band power with all clinical assessments (**A**), MCCB overall composite T-score (**B**), and PANSS total score (**C**) at baseline. + Correlation coefficient ≥0.3 and *p* < 0.05; − correlation coefficient ≤ −0.3 and *p* < 0.05. ASSR auditory steady-state response, EEG electroencephalography, LNS letter-number span, MATRICS Measurement and Treatment Research to Improve Cognition in Schizophrenia, MCCB MATRICS Consensus Cognitive Battery, MMN mismatch negativity, NAB neuropsychological assessment battery, PANSS positive and negative syndrome scale, PLF phase-locking factor, TMTA Trail Making Test Part A, WMS-III SS Wechsler Memory Scale 3rd edition, Spatial Span.

Baseline EEG data are shown in Supplementary Table 2. Comparisons of baseline EEG data with baseline clinical assessments demonstrated significant positive correlations between ASSR PLF and MCCB overall composite T-scores and MCCB neurocognitive composite T-scores, in addition to weak-to-moderate positive correlations with selected MCCB subdomains (LNS, WMS-III SS, NAB mazes, and TMT-A T-score) (Fig. 1A, B; Supplementary Table 3). Further, ASSR evoked and induced power were positively, albeit not significantly, correlated with MCCB overall T-scores, neurocognitive composite T-scores, and the four MCCB subdomain T-scores (Fig. 1A; Supplementary Table 3).

For MMN amplitude, the frequency, and frequency + duration deviants were significantly positively correlated with PANSS general psychopathology and PANSS total scores (Fig. 1A, C; Supplementary Table 3), indicating that a worsening in these clinical scores was associated with disease-related deficits in MMN. In general, all MMN amplitude deviants showed positive correlations with PANSS total score and all three subscales, although these differences were not always statistically significant and were less pronounced for the duration deviant than for other deviants (Fig. 1A; Supplementary Table 3). Conversely, MMN amplitude deviants showed either very weak positive or weak-to-moderate negative correlations with MCCB overall and neurocognitive composite T-scores, as well as the MCCB subdomain T-scores (Fig. 1A; Supplementary Table 3). Interactions between gamma power and clinical assessments at baseline demonstrated the weakest correlations overall (Fig. 1A; Supplementary Table 3).

### EEG parameters as treatment response biomarkers

CfB at EoT for MMN amplitudes, ASSR parameters, and gamma power in the placebo group and the combined 10 + 25 mg dose groups is shown in Table 2, while absolute values for EEG parameters at EoT are shown in Supplementary Table 4. Typically, a negative CfB value for MMN would indicate an improvement in MMN amplitude and a positive CfB would indicate a worsening of this parameter, while the opposite applies for ASSR and resting state gamma parameters. Notably, however, only slight differences in MMN frequency amplitude and ASSR evoked power were observed between the treated group versus placebo, though these were not deemed to be clinically meaningful (Table 2). Grand average waveforms for MMN amplitude (duration) and ASSR PLF for the combined iclepertin 10 + 25 mg dose groups (Supplementary Fig. 3) or placebo (Supplementary Fig. 4) further illustrated that there was no treatment response following administration of iclepertin.

**Table 2:** CfB in selected EEG parameters for the Fz electrode at EoT.

	MMN amplitude			ASSR^a^ (40 Hz stimulation)			Resting state power in gamma band	
	Duration (µV)	Frequency (µV)	Duration + frequency (µV)	Induced power (dB)	Evoked power (dB)	Phase-locking factor	Absolute power (µV2)	Relative power (%)
Total patients (*N* = 79)								
N^b^	47	46	47	37			57	
Mean	−0.02	0.17	−0.10	0.15	−0.09	−0.00	−0.02	−0.23
SEM	0.21	0.20	0.25	0.20	1.20	0.01	0.26	0.25
SD	1.41	1.33	1.70	1.20	7.27	0.07	1.93	1.88
Min	−2.54	-3.21	−4.89	−1.87	−12.21	−0.13	−6.21	−11.52
Max	3.31	3.23	3.28	4.03	13.02	0.19	10.29	4.81
Placebo (*N* = 20)								
N^b^	12			10			15	
Mean	−0.22	0.68	0.29	0.26	0.79	0.01	−0.76	−0.46
SEM	0.40	0.47	0.61	0.45	2.25	0.03	0.28	0.25
SD	1.38	1.62	2.11	1.42	7.13	0.08	1.10	0.96
Min	−2.18	−2.17	-3.82	−1.87	−10.91	−0.07	−3.97	−3.20
Max	1.70	2.94	3.28	3.41	11.83	0.19	0.83	1.23
Iclepertin 10 + 25 mg (*N* = 35)								
N^b^	21			15			26	
Mean	−0.01	0.33	-0.08	0.01	−2.09	−0.01	0.24	−0.43
SEM	0.30	0.23	0.40	0.20	1.78	0.02	0.50	0.47
SD	1.36	1.05	1.84	0.76	6.90	0.06	2.56	2.41
Min	−2.54	−1.59	−4.89	−1.53	−12.21	−0.13	−6.21	−11.52
Max	1.72	3.23	2.79	0.99	12.33	0.09	10.29	2.15

*ASSR* auditory steady-state response, *CfB*change from baseline; *EEG* electroencephalography; *EoT* end of treatment, *MMN* mismatch negativity, *SD* standard deviation; *SEM* standard error of the meanASSR, auditory steady-state response; *CfB* change from baseline, *EEG* electroencephalography, *EoT* end of treatment, *MMN* mismatch negativity, *SD* standard deviation, *SEM* standard error of the mean.

^a^Baseline-corrected.

^b^Number of patients with analyzable data for each EEG parameter.

**Fig. 2:**
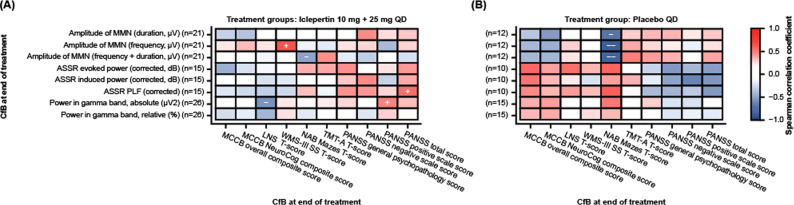
EEG parameters as treatment response biomarkers. Spearman correlation of CfB in EEG parameters with CfB in clinical assessments in combined 10 + 25 mg dose groups (**A**) and placebo group (**B**). + Correlation coefficient ≥0.3 and *p* < 0.05; − correlation coefficient ≤ −0.7 and *p* < 0.05; − correlation coefficient ≤ −0.3 and *p* < 0.05. ASSR auditory steadystate response, CfB change from baseline, EEG electroencephalography, LNS letter-number span, MATRICS Measurement and Treatment Research to Improve Cognition in Schizophrenia, MCCB MATRICS Consensus Cognitive Battery, MMN mismatch negativity, NAB neuropsychological assessment battery, PANSS positive and negative syndrome scale, PLF phase-locking factor, TMTA Trail Making Test Part A, WMS-III SS Wechsler Memory Scale 3rd edition, Spatial Span.

The pattern of correlation between CfB in EEG and clinical scores at EoT is shown in Fig. 2. There were no significant correlations between CfB for either MCCB overall or neurocognitive composite T-scores and CfB for any of the measured EEG parameters following treatment with iclepertin; however, ASSR PLF, and absolute gamma power were significantly correlated with PANSS total score and PANSS positive scale score, respectively (Fig. 2A; Supplementary Table 5). For the individual MCCB tests, CfB in MMN amplitude frequency showed a moderate positive correlation with CfB in WMS-III SS T-score in the iclepertin 10 + 25 mg combined treatment group (Fig. 2A; Supplementary Table 5), but not in the placebo group (Fig. 2B; Supplementary Table 5). In addition, CfB in LNS T-scores were significantly negatively correlated with absolute gamma power (*p* < 0.05), while NAB mazes T-scores were significantly negatively correlated with MMN amplitude frequency + duration (*p* < 0.05) (data not shown). However, for the 10 + 25 mg dose group there was no clear pattern of correlations observed between clinical assessments and groups of EEG parameters that may be expected to demonstrate similar changes (e.g., each of the three MMN amplitude deviants, or each of the ASSR parameters).

Notably, a similar pattern of correlations was observed in the overall study population at baseline (Fig. 1A) and CfB at EoT for the placebo group (Fig. 2B; Supplementary Table 5).

### EEG parameters as predictive biomarkers of treatment response

**Fig. 3:**
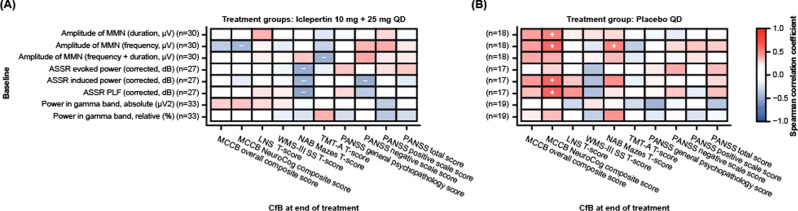
EEG parameters as predictive biomarkers. Spearman correlation of EEG parameters at baseline with CfB in clinical assessments in the combined 10 + 25 mg dose groups (**A**) and placebo group (**B**). + Correlation coefficient ≥0.3 and *p* < 0.05; − correlation coefficient ≤ −0.3 and *p* < 0.05. ASSR auditory steady-state response, CfB change from baseline, EEG electroencephalography, LNS letter-number span, MATRICS Measurement and Treatment Research to Improve Cognition in Schizophrenia, MCCB MATRICS Consensus Cognitive Battery, MMN mismatch negativity, NAB neuropsychological assessment battery, PANSS positive and negative syndrome scale, PLF phase-locking factor, TMT-A Trail Making Test Part A, WMS-III SS Wechsler Memory Scale 3rd edition, spatial span.

Overall, correlations between baseline EEG parameters and CfB in clinical assessments at EoT were generally weak for the iclepertin 10 + 25 mg group (Fig. 3A; Supplementary Table 6). However, all ASSR parameters at baseline were significantly negatively correlated with CfB in NAB mazes T-score for the iclepertin 10 + 25 mg group, while ASSR-induced power was significantly negatively correlated with PANSS negative subscale score among this group at EoT (Fig. 3A; Supplementary Table 6). Further, MMN amplitude frequency and frequency + duration deviants were significantly negatively correlated with MCCB neurocognitive composite T-score and TMT-A T-score, respectively (Fig. 3A; Supplementary Table 6). In the placebo group, baseline MMN amplitude frequency deviants MMN amplitude duration deviants, ASSR induced power, and ASSR PLF were significantly positively correlated with CfB in MCCB neurocognitive composite T-score (Fig. 3B; Supplementary Table 6).

## Discussion

Data from this iclepertin substudy suggest that certain EEG parameters may represent potentially useful neurophysiological biomarkers for assessing schizophrenia-related cognitive impairment and symptoms. In particular, the baseline correlation of ASSR parameters with MCCB scores and MMN amplitude with PANSS warrants further evaluation, since these data suggest a correlation between EEG parameters and clinical assessments covering all three domains of schizophrenia symptoms (positive, negative, and cognitive). Furthermore, the correlation of EEG parameters and clinical assessments at EoT among the placebo group closely reflected findings reported for the total group at baseline, further supporting the utility of respective EEG parameters as neurophysiological markers of schizophrenia-related deficits. Moreover, data from this study raise the possibility that individual EEG parameters may represent specific biomarkers of particular neurophysiological deficits or symptoms associated with schizophrenia. For example, ASSR measures were consistently positively correlated with both MCCB overall and neurocognitive composite T-scores, while MMN amplitude deviants were consistently positively correlated with PANSS scores. It should be noted, however, that the current study was designed for hypothesis generation, and no adjustments for multiple comparisons were made, meaning that the study may be vulnerable to chance findings. Therefore, these initial results require further validation from future studies.

Nonetheless, our findings are consistent with previous studies describing the correlation of deficits in ASSR parameters with deficits in clinical assessments measuring cognition in patients with schizophrenia [22, 34]. Specifically, Sun et al. described a significant correlation between PLF and cognitive scores of reasoning and problem solving, in line with our findings [34]. However, the current study employed a larger dataset than previously published [34], providing further strength to our initial findings. In a larger study, Koshiyama and colleagues reported correlations between deficits in gamma-band ASSR with working memory deficits and impairment in daily functioning, and between MMN deficits with reduced verbal learning and impaired functioning [22]. Previously published reports from single-center studies also indicate a correlation between deficits in MMN amplitude with positive [35, 36] and negative [36, 37] symptoms associated with schizophrenia, similar to those reported here.

Deficits in MMN and ASSR parameters among patients with schizophrenia are considered to be caused mainly by aberrant NMDAR-signaling [21], supporting the potential use of these EEG parameters as biomarkers for monitoring treatment response to NMDAR-modulating agents. This hypothesis is supported by previous studies, unraveling a correlation of EEG markers with cognitive read-outs and positive and negative symptoms in response to NMDAR-modulating treatment. For example, Greenwood and colleagues detected an attenuation of deficits in MMN, coinciding with improvements in PANSS-Total, PANSS-Negative, and PANSS-General scores among patients with schizophrenia who were treated with the NMDAR-co-agonist glycine [23]. Similarly, Kantrowitz and colleagues detected an attenuation in MMN deficits accompanying an improvement of clinical symptoms when patients with schizophrenia were treated with the NMDAR co-agonist, D-serine [24]. Treatment with the GlyT1 inhibitor, bitopertin [25], did not significantly effect EEG parameters in patients with schizophrenia, though this could possibly be explained by the small sample size (17 on active, 12 on placebo) or the minor clinical effects of bitopertin in improving positive and negative symptoms in a Phase III trial [27].

However, despite the consistent patterns of correlations seen for some EEG parameters and clinical assessments at baseline, most correlations observed in the current substudy following treatment with iclepertin were weak to moderate, suggesting limited potential of these EEG parameters as biomarkers of treatment response or as predictors of treatment response to iclepertin in the present study. Potential reasons for the modest correlations observed in this substudy may include high levels of EEG variability resulting from the multicenter design and varying levels of expertize across study sites in conducting pharmaco-EEG studies. However, the inclusion of multiple sites could also be considered a strength of the study, as this design may be expected to improve replicability compared with single-site studies. It should also be noted that although the overall study population was relatively large, there were limited numbers of patients in the individual dose groups in this substudy, resulting in high levels of variation in the demographic and clinical characteristics reported here. This variability may have limited the detection of significant correlations between EEG parameters and clinical assessments. The use of multiple study sites with larger patient numbers would ensure the inclusion of a broader study population that is more reflective of the real-world patient population.

The observed correlations may also have been influenced by the inherent differences between cognitive assessments and objective biomarkers in their reliability for detecting individual variability. This effect, described in the literature as the “reliability paradox”, limits the correlation that can be observed between two factors when low between-subject variability causes low reliability for individual differences for each cognitive paradigm [38]. It is also possible that concomitant medication use may have affected our findings. For example, patients with schizophrenia receiving atypical antipsychotics had significantly enhanced 40 Hz synchronization compared with those taking conventional antipsychotics [39]. Similarly, in patients with schizophrenia treated with atypical antipsychotics 30–50 Hz ASSR response was normalized to a level similar to healthy controls, while ASSR deficits persisted among untreated patients [40]. However, the evidence for positive effects of antipsychotic medications on EEG deficits remains inconclusive; for example, another study reported no effect of antipsychotic use (conventional or atypical) on ASSR among patients with schizophrenia [41]. The uncompetitive NMDA antagonist memantine has also been shown to alter cortical E/I balance [42]; memantine is typically used to treat Alzheimer’s disease [42], though it may also be effective as a pro-cognitive adjunctive therapy in patients with schizophrenia [43]. Furthermore, memantine can normalize gamma power deficits in patients with schizophrenia [44]. Taken together, these findings suggest that the sensory and processing deficits typically observed in patients with schizophrenia [4–6] could have been normalized to some extent by background medication use among our cohort, which may have contributed to the observation of only modest correlations between EEG parameters and clinical assessments in this study. Thus, with sites specializing in EEG in patients and further technical refinement, it is conceivable that EEG parameters could potentially be useful as biomarkers of treatment response.

Despite these potential limitations, the grand average waveforms reported here showed limited variation at baseline and EoT, supporting the integrity of our data and indicating that further study on the basis of our initial findings may be warranted.

## Conclusion

This substudy provides preliminary results that encourage the use of EEG biomarkers to monitor neurophysiological changes associated with CIAS. Further refinement of data processing techniques, along with limiting data collection to sites experienced with conducting pharmaco-EEG studies, would be expected to help establish EEG parameters as useful biomarkers in schizophrenia and other central nervous system indications in the future.

## Supplementary information

Supplementary Material

Supplementary Figure 1

Supplementary Figure 2

Supplementary Figure 3

Supplementary Figure 4
