# Supplementary material for: Quantitative electroencephalography parameters as neurophysiological biomarkers of schizophrenia-related deficits: A Phase II substudy of patients treated with iclepertin (BI 425809)
Source: Transl Psychiatry. 2022 Aug 11;12:329. doi: 10.1038/s41398-022-02096-5 (PMC9372178; doi:10.1038/s41398-022-02096-5)
Supplement: Supplementary file 1 — Supplementary Material [file 41398_2022_2096_MOESM1_ESM.docx]

**SUPPLEMENTARY MATERIAL**

**Quantitative electroencephalography parameters as neurophysiological biomarkers of schizophrenia-related deficits: A Phase II substudy of patients treated with iclepertin (BI 425809)**

Christian Schultheis^1^, Holger Rosenbrock^1^, Salome Rebecca Mack^1^, Richard Vinisko^2^, Niklas Schuelert^1^, Andrea Plano^1^, Sigurd D. Süssmuth^3^

^1^Boehringer Ingelheim Pharma GmbH & Co. KG, Biberach an der Riss, Germany; ^2^Boehringer Ingelheim Pharmaceuticals Inc., Ridgefield, CT, USA;
^3^Boehringer Ingelheim International GmbH, Biberach an der Riss, Germany

**Corresponding author:** Christian Schultheis

**Address:** Department of Translational Medicine and Clinical Pharmacology, Boehringer Ingelheim Pharma GmbH & Co. KG, 88397 Biberach an der Riss, Germany

**Email:** [christian.schultheis@boehringer-ingelheim.com](mailto:christian.schultheis@boehringer-ingelheim.com)

**Tel:** +49 (7351) 54-7685

**Target journal:** *Translational Psychiatry*

**SUPPLEMENTARY FIGURES**

**Supplementary Figure 1. Study design^a^**

^a^First EEG measurements were taken 14 days prior to study commencing (Day 1); final EEG measurements were taken 7 days prior to EoT (Week 12). Number of patients shown for each dose group indicates the number randomized in the EEG substudy.

EEG, electroencephalography; EoT, end of treatment

**Supplementary Figure 2. Patient disposition in EEG substudy**

^a^Data that did not meet predefined quality control criteria were excluded

EEG, electroencephalography

**Supplementary Figure 3. Grand average waveform at baseline and EoT for MMN duration deviant (A), and PLF (B) for the combined 10 + 25 mg dose groups**

For MMN duration deviant, the difference wave (dDur-STD) is depicted as a solid line; the standard wave is depicted as a dashed line. N=30 for MMN at baseline; N=26 for MMN at EoT. N=26 for PLF at baseline; N=20 for PLF at EoT. Data shown as mean
+/-SEM

dDur-STD, deviant duration minus standard; EoT, end of treatment; MNN, mismatch negativity; PLF, phase-locking factor; STD, standard; SEM, standard error of the mean

**Supplementary Figure 4. Grand average waveform at baseline and EoT for MMN duration deviant (A), and PLF (B) for the placebo group**

For MMN duration deviant, the difference wave (dDur-STD) is depicted as a solid line; the standard wave is depicted as a dashed line. N=30 for MMN at baseline; N=26 for MMN at EoT. N=26 for PLF at baseline; N=20 for PLF at EoT. Data shown as mean +/-SEM

dDur-STD, deviant duration minus standard; EoT, end of treatment; MNN, mismatch negativity; PLF, phase-locking factor; STD, standard; SEM, standard error of the mean

**SUPPLEMENTARY TABLES**

**Supplementary Table 1. Selected eligibility criteria for the iclepertin parent trial^a^ and EEG substudy**

| **Inclusion criteria** | **Exclusion criteria** |
| --- | --- |
| - Male or female patients aged 18–50 years - DSM-5 criteria diagnosis of schizophrenia - Medically stable over the prior 4 weeks  (up to two antipsychotics, typical or atypical, except for clozapine), and psychiatrically stable without symptom exacerbation within 3 months prior to study randomization - Informed consent to take part in parent study as well as EEG substudy - Educational level sufficient to comply with protocol procedures - Willingness and ability to comply with the requirements for EEG procedures | - Diagnosis of any other major psychiatric disorder, including current drug abuse disorder - Diseases of the CNS that may impact the assessment of the cognitive tests - Participation in any formal remediation program - Any circumstance or condition that could compromise the validity of cognitive outcome measures, or interfere with EEG procedures used in this substudy (e.g., use of hearing aids) |

^a^Full eligibility criteria for the parent trial have been previously published [31]

CNS, central nervous system; DSM-5, Diagnostic and Statistical Manual of Mental Disorders, 5th edition; EEG, electroencephalograph

**Supplementary Table 2. Selected EEG parameters for the Fz electrode at baseline**

|  | **MMN Amplitude** | | | **ASSR^a^ (40 Hz stimulation)** | | | | | **Resting state power in gamma band** | | |
| --- | --- | --- | --- | --- | --- | --- | --- | --- | --- | --- | --- |
|  | **Duration  (µV)** | **Frequency (µV)** | **Duration + frequency (µV)** | **Induced power (dB)** | **Evoked power (dB)** | | **Phase-locking factor** | | **Absolute power (µV2)** | | **Relative power  (%)** |
| **Total patients (N=79)** | | | | | | | | | | | |
| **N^b^** | **69** | | | **62** | | | | | **76** | | |
| **Mean** | -3.18 | -2.69 | -3.41 | 1.77 | | 16.05 | | 0.15 | 2.82 | 2.65 | |
| **SEM** | 0.21 | 0.18 | 0.21 | 0.19 | | 0.71 | | 0.01 | 0.28 | 0.35 | |
| **SD** | 1.73 | 1.48 | 1.73 | 1.53 | | 5.62 | | 0.07 | 2.44 | 3.04 | |
| **Min** | -9.32 | -6.16 | -8.48 | 0.15 | | 5.02 | | 0.04 | 0.69 | 0.43 | |
| **Max** | -0.01 | -0.24 | 0.55 | 7.65 | | 34.33 | | 0.33 | 16.60 | 22.94 | |
| **Placebo (N=20)** | | | | | | | | | | | |
| **N^b^** |  | **18** |  |  | | **17** | |  | **19** | | |
| **Mean** | -2.96 | -3.11 | -3.61 | 1.95 | | 16.12 | | 0.15 | 2.83 | 2.50 | |
| **SEM** | 0.33 | 0.36 | 0.43 | 0.49 | | 1.46 | | 0.02 | 0.44 | 0.49 | |
| **SD** | 1.39 | 1.53 | 1.82 | 2.01 | | 6.02 | | 0.07 | 1.91 | 2.12 | |
| **Min** | -6.52 | -5.96 | -8.48 | 0.24 | | 7.28 | | 0.05 | 0.94 | 0.73 | |
| **Max** | -1.39 | -0.93 | -0.83 | 7.65 | | 34.33 | | 0.33 | 9.27 | 9.80 | |
| **Iclepertin 10 + 25 mg (N=35)** | | | | | | | | | | | |
| **N^b^** |  | **30** |  |  | | **27** | |  | **33** | | |
| **Mean** | -3.02 | -2.50 | -3.13 | 1.91 | | 17.14 | | 0.16 | 3.10 | 3.28 | |
| **SEM** | 0.29 | 0.26 | 0.31 | 0.27 | | 1.07 | | 0.01 | 0.54 | 0.72 | |
| **SD** | 1.59 | 1.44 | 1.69 | 1.40 | | 5.57 | | 0.07 | 3.07 | 4.15 | |
| **Min** | -6.56 | -5.92 | -6.79 | 0.15 | | 7.66 | | 0.04 | 0.98 | 0.74 | |
| **Max** | -0.27 | -0.31 | 0.55 | 5.42 | | 29.48 | | 0.32 | 16.59 | 22.94 | |

^a^Baseline-corrected. ^b^Number of patients with analyzable data for each EEG parameter.

ASSR, auditory steady-state response; EEG, electroencephalography; MMN, mismatch negativity; SD, standard deviation; SEM, standard error of the mean

**Supplementary Table 3. EEG parameters as neurophysiological biomarkers: Spearman correlation of EEG parameters at baseline with baseline clinical assessments for overall treated group**

|  | **MCCB Overall Composite T-Score** | **MCCB NeuroCog Composite T-Score** | **LNS  T-Score** | **WMS-III SS T-Score** | **NAB Mazes  T-Score** | **TMT  T-Score** | **PANSS General Psycho-pathology Score** | **PANSS Negative Scale Score** | **PANSS Positive Scale Score** | **PANSS Total Score** |
| --- | --- | --- | --- | --- | --- | --- | --- | --- | --- | --- |
| **MMN amplitude, Spearman correlation** | | | | | | | | | | |
| Duration (µV) | -0.07 | 0.01 | -0.20 | -0.06 | -0.02 | -0.13 | 0.19 | 0.11 | 0.05 | 0.16 |
| Frequency (µV) | -0.06 | -0.02 | -0.07 | -0.12 | 0.07 | 0.03 | 0.36* | 0.28 | 0.27 | 0.35* |
| Duration + frequency (µV) | -0.24 | -0.21 | -0.32* | -0.32* | -0.04 | -0.09 | 0.32* | 0.30 | 0.20 | 0.30* |
| **ASSR at 40 Hz, Spearman correlation** | | | | | | | | | | |
| Evoked power (dB) | 0.26 | 0.23 | 0.14 | 0.06 | 0.17 | 0.21 | 0.04 | 0.05 | 0.18 | 0.08 |
| Induced power (dB) | 0.16 | 0.13 | 0.05 | 0.11 | 0.02 | 0.17 | -0.01 | -0.01 | 0.03 | 0.01 |
| Phase-locking factor | 0.33* | 0.34* | 0.22 | 0.22 | 0.21 | 0.24 | 0.16 | 0.00 | 0.16 | 0.15 |
| **Resting state power in gamma band, Spearman correlation** | | | | | | | | | | |
| Absolute power (µV2) | -0.22 | -0.22 | -0.24 | -0.17 | -0.13 | -0.04 | -0.22 | -0.04 | 0.03 | -0.03 |
| Relative power (%) | -0.08 | -0.06 | -0.36* | -0.14 | -0.11 | -0.13 | -0.09 | -0.05 | -0.05 | -0.08 |

*Correlation coefficient is ≥0.3, or ≤−0.3 and p-value <0.05

ASSR, auditory steady-state response; EEG, electroencephalography; LNS, Letter-Number Span; MATRICS, Measurement and Treatment Research to Improve Cognition in Schizophrenia; MCCB, MATRICS Consensus Cognitive Battery; MMN, mismatch negativity; NAB, Neuropsychological Assessment Battery; PANSS, Positive and Negative Syndrome Scale; PLF, phase-locking factor; TMT-A, Trail Making Test Part A; WMS-III SS, Wechsler Memory Scale 3rd edition, Spatial Span

**Supplementary Table 4. Selected EEG parameters for the Fz electrode at EoT**

|  | **MMN Amplitude** | | | | **ASSR^a^ (40 Hz stimulation)** | | | | | **Resting state power in gamma band** | | |
| --- | --- | --- | --- | --- | --- | --- | --- | --- | --- | --- | --- | --- |
|  | **Duration  (µV)** | | **Frequency (µV)** | **Duration + frequency (µV)** | **Induced power (dB)** | **Evoked power (dB)** | | **Phase-locking factor** | | **Absolute power (µV2)** | | **Relative power  (%)** |
| **Total patients (N=79)** | | | | | | | | | | | | |
| **N^b^** | **52** | **51** | | **52** | **44** | | | | | **58** | | |
| **Mean** | -3.46 | | -2.80 | -3.77 | 1.63 | | 15.09 | | 0.14 | 2.91 | 2.55 | |
| **SEM** | 0.26 | | 0.20 | 0.24 | 0.23 | | 0.85 | | 0.01 | 0.41 | 0.35 | |
| **SD** | 1.85 | | 1.39 | 1.75 | 1.52 | | 5.63 | | 0.08 | 3.12 | 2.66 | |
| **Min** | -8.82 | | -6.32 | -8.79 | -0.41 | | 4.94 | | 0.02 | 0.44 | 0.52 | |
| **Max** | 0.95 | | -0.05 | -0.19 | 6.34 | | 26.73 | | 0.36 | 21.16 | 15.60 | |
| **Placebo (N=20)** | | | | | | | | | | | | |
| **N^b^** |  | | **13** |  |  | | **13** | |  | **16** | | |
| **Mean** | -3.45 | | -2.83 | -3.67 | 1.54 | | 15.39 | | 0.14 | 2.26 | 2.45 | |
| **SEM** | 0.22 | | 0.29 | 0.35 | 0.38 | | 1.35 | | 0.02 | 0.29 | 0.43 | |
| **SD** | 0.80 | | 1.04 | 1.28 | 1.37 | | 4.88 | | 0.08 | 1.14 | 1.73 | |
| **Min** | -4.87 | | -5.27 | -5.28 | 0.21 | | 6.62 | | 0.06 | 0.98 | 0.62 | |
| **Max** | -2.08 | | -1.11 | -1.19 | 5.05 | | 26.73 | | 0.36 | 5.30 | 6.60 | |
| **Iclepertin 10 + 25 mg (N=35)** | | | | | | | | | | | | |
| **N^b^** |  | | **23** |  |  | | **18** | |  | **26** | | |
| **Mean** | -3.35 | | -2.40 | -3.70 | 1.57 | | 14.17 | | 0.13 | 3.46 | 2.97 | |
| **SEM** | 0.38 | | 0.30 | 0.45 | 0.35 | | 1.36 | | 0.02 | 0.80 | 0.65 | |
| **SD** | 1.82 | | 1.42 | 2.14 | 1.49 | | 5.75 | | 0.08 | 4.08 | 3.34 | |
| **Min** | -7.39 | | -6.32 | -8.79 | -0.24 | | 4.94 | | 0.02 | 0.97 | 0.81 | |
| **Max** | -0.17 | | -0.05 | -0.19 | 6.02 | | 25.95 | | 0.33 | 21.16 | 15.60 | |

^a^Baseline-corrected. ^b^Number of patients with analyzable data for each EEG parameter.

ASSR, auditory steady-state response; EEG, electroencephalography; EoT, end of treatment; MMN, mismatch negativity; SD, standard deviation; SEM, standard error of the mean

**Supplementary Table 5. EEG parameters as treatment response biomarkers: Spearman correlation of CfB in EEG parameters with CfB in clinical assessments in combined 10 + 25 mg dose group, versus placebo**

|  | **MCCB Overall Composite T-Score** | **MCCB NeuroCog Composite T-Score** | **LNS  T-Score** | **WMS-III SS T-Score** | **NAB Mazes  T-Score** | **TMT  T-Score** | **PANSS General Psycho-pathology Score** | **PANSS Negative Scale Score** | **PANSS Positive Scale Score** | **PANSS Total Score** |
| --- | --- | --- | --- | --- | --- | --- | --- | --- | --- | --- |
| **Iclepertin 10 + 25 mg** | | | | | | | | | | |
| **MMN amplitude, Spearman correlation** | | | | | | | | | | |
| Duration (µV) | -0.25 | -0.32 | -0.03 | 0.05 | -0.04 | 0.06 | 0.05 | 0.43 | 0.16 | 0.23 |
| Frequency (µV) | 0.12 | 0.27 | 0.14 | 0.66* | -0.12 | -0.15 | 0.15 | 0.19 | -0.36 | 0.20 |
| Duration + frequency (µV) | -0.05 | -0.06 | -0.11 | 0.03 | -0.46* | 0.42 | -0.15 | -0.27 | 0.01 | -0.20 |
| **ASSR at 40 Hz, Spearman correlation** | | | | | | | | | | |
| Evoked power (dB) | -0.39 | -0.19 | -0.09 | -0.06 | 0.30 | 0.33 | 0.41 | 0.07 | 0.15 | 0.39 |
| Induced power (dB) | -0.01 | -0.01 | -0.19 | -0.16 | -0.01 | 0.04 | 0.23 | 0.44 | -0.25 | 0.33 |
| Phase-locking factor | -0.17 | -0.22 | -0.26 | -0.23 | 0.26 | -0.11 | 0.41 | 0.40 | 0.18 | 0.54* |
| **Resting state power in gamma band, Spearman correlation** | | | | | | | | | | |
| Absolute power (µV2) | -0.07 | 0.04 | -0.52* | 0.13 | 0.07 | 0.11 | 0.18 | 0.01 | 0.46* | 0.27 |
| Relative power (%) | -0.21 | -0.15 | -0.38 | 0.16 | -0.16 | 0.02 | 0.07 | -0.17 | 0.21 | 0.07 |
| **Placebo** | | | | | | | | | | |
| **MMN amplitude, Spearman correlation** | | | | | | | | | | |
| Duration (µV) | -0.30 | -0.48 | 0.01 | -0.04 | -0.67* | 0.21 | 0.28 | 0.29 | 0.12 | 0.23 |
| Frequency (µV) | -0.36 | -0.40 | 0.20 | 0.02 | -0.78** | 0.34 | 0.18 | 0.11 | -0.13 | 0.15 |
| Duration + frequency (µV) | -0.37 | -0.38 | 0.14 | -0.05 | -0.76** | 0.48 | 0.38 | 0.31 | -0.05 | 0.34 |
| **ASSR at 40 Hz, Spearman correlation** | | | | | | | | | | |
| Evoked power (dB) | 0.57 | 0.36 | 0.54 | 0.29 | 0.58 | 0.01 | -0.24 | -0.49 | -0.36 | -0.47 |
| Induced power (dB) | 0.51 | 0.28 | 0.12 | -0.19 | 0.35 | 0.30 | -0.15 | -0.46 | -0.60 | -0.50 |
| Phase-locking factor | 0.44 | 0.29 | 0.38 | 0.26 | 0.60 | 0.08 | -0.35 | -0.38 | -0.29 | -0.49 |
| **Resting state power in gamma band, Spearman correlation** | | | | | | | | | | |
| Absolute power (µV2) | 0.31 | 0.37 | 0.03 | -0.02 | 0.50 | -0.03 | 0.20 | -0.18 | -0.18 | -0.03 |
| Relative power (%) | 0.27 | 0.33 | 0.02 | 0.16 | 0.29 | 0.05 | 0.02 | -0.18 | -0.13 | -0.15 |

*Correlation coefficient is ≥0.3, or ≤−0.3 and p-value<0.05; **correlation coefficient is ≥0.7 or ≤−0.7 and p-value <0.05

ASSR, auditory steady-state response; CfB, change from baseline; EEG, electroencephalography; LNS, Letter-Number Span; MATRICS, Measurement and Treatment Research to Improve Cognition in Schizophrenia; MCCB, MATRICS Consensus Cognitive Battery; MMN, mismatch negativity; NAB, Neuropsychological Assessment Battery; PANSS, Positive and Negative Syndrome Scale; PLF, phase-locking factor; TMT-A, Trail Making Test Part A; WMS-III SS, Wechsler Memory Scale 3rd edition, Spatial Span

**Supplementary Table 6. EEG parameters as predictive biomarkers: Spearman correlation of EEG parameters at baseline with CfB in clinical assessments in the combined 10 + 25 mg dose group, or placebo**

|  | **MCCB Overall Composite T-Score** | **MCCB NeuroCog Composite T-Score** | **LNS  T-Score** | **WMS-III SS T-Score** | **NAB Mazes  T-Score** | **TMT  T-Score** | **PANSS General Psycho-pathology Score** | **PANSS Negative Scale Score** | **PANSS Positive Scale Score** | **PANSS Total Score** |
| --- | --- | --- | --- | --- | --- | --- | --- | --- | --- | --- |
| **Iclepertin 10 + 25 mg** | | | | | | | | | | |
| **MMN amplitude, Spearman correlation** | | | | | | | | | | |
| Duration (µV) | 0.03 | -0.06 | 0.32 | -0.02 | 0.07 | -0.18 | -0.09 | -0.04 | 0.19 | -0.07 |
| Frequency (µV) | -0.30 | -0.40* | 0.09 | -0.15 | -0.00 | -0.27 | -0.01 | 0.31 | 0.34 | 0.13 |
| Duration + frequency (µV) | -0.07 | -0.08 | 0.12 | 0.08 | 0.25 | -0.44* | 0.03 | 0.36 | 0.14 | 0.12 |
| **ASSR at 40 Hz, Spearman correlation** | | | | | | | | | | |
| Evoked power (dB) | -0.01 | -0.02 | -0.00 | -0.11 | -0.44* | -0.13 | 0.26 | 0.01 | 0.17 | 0.24 |
| Induced power (dB) | -0.05 | -0.15 | -0.03 | -0.09 | -0.43* | -0.03 | 0.05 | -0.45* | 0.02 | -0.16 |
| Phase-locking factor | -0.01 | -0.04 | 0.14 | 0.11 | -0.48* | -0.03 | 0.19 | -0.31 | -0.08 | -0.03 |
| **Resting state power in gamma band, Spearman correlation** | | | | | | | | | | |
| Absolute power (µV2) | 0.22 | 0.26 | 0.19 | 0.13 | -0.09 | -0.06 | -0.01 | 0.06 | -0.30 | -0.07 |
| Relative power (%) | 0.01 | 0.00 | -0.02 | -0.14 | -0.19 | 0.33 | -0.22 | -0.06 | -0.31 | -0.23 |
| **Placebo** | | | | | | | | | | |
| **MMN amplitude, Spearman correlation** | | | | | | | | | | |
| Duration (µV) | 0.39 | 0.51* | 0.10 | 0.15 | 0.19 | 0.03 | -0.10 | -0.15 | 0.02 | -0.05 |
| Frequency (µV) | 0.40 | 0.62* | -0.03 | -0.17 | 0.51* | -0.30 | 0.03 | 0.19 | 0.26 | 0.23 |
| Duration + frequency (µV) | 0.36 | 0.33 | -0.06 | 0.09 | 0.29 | -0.17 | -0.06 | -0.10 | 0.15 | -0.01 |
| **ASSR at 40 Hz, Spearman correlation** | | | | | | | | | | |
| Evoked power (dB) | -0.09 | 0.20 | 0.05 | -0.35 | 0.02 | 0.04 | 0.13 | 0.36 | 0.00 | 0.28 |
| Induced power (dB) | 0.41 | 0.62* | 0.25 | -0.36 | 0.46 | -0.13 | 0.09 | 0.04 | -0.07 | 0.18 |
| Phase-locking factor | 0.29 | 0.64* | 0.43 | -0.35 | 0.14 | 0.08 | 0.09 | 0.25 | -0.15 | 0.27 |
| **Resting state power in gamma band, Spearman correlation** | | | | | | | | | | |
| Absolute power (µV2) | -0.04 | -0.06 | -0.24 | 0.15 | 0.07 | -0.23 | -0.41 | -0.10 | -0.04 | -0.33 |
| Relative power (%) | 0.11 | 0.28 | -0.01 | -0.28 | 0.44 | -0.08 | -0.16 | 0.08 | -0.20 | -0.10 |

*Correlation coefficient is ≥0.3, or ≤−0.3 and p-value <0.05

ASSR, auditory steady-state response; CfB, change from baseline; EEG, electroencephalography; LNS, Letter-Number Span; MATRICS, Measurement and Treatment Research to Improve Cognition in Schizophrenia; MCCB, MATRICS Consensus Cognitive Battery; MMN, mismatch negativity; NAB, Neuropsychological Assessment Battery; PANSS, Positive and Negative Syndrome Scale; PLF, phase-locking factor; TMT-A, Trail Making Test Part A; WMS-III SS, Wechsler Memory Scale 3rd edition, Spatial Span
