# Supplementary figures and images for: Quantitative electroencephalography parameters as neurophysiological biomarkers of schizophrenia-related deficits: A Phase II substudy of patients treated with iclepertin (BI 425809)
Source: Transl Psychiatry. 2022 Aug 11;12:329. doi: 10.1038/s41398-022-02096-5 (PMC9372178; doi:10.1038/s41398-022-02096-5)

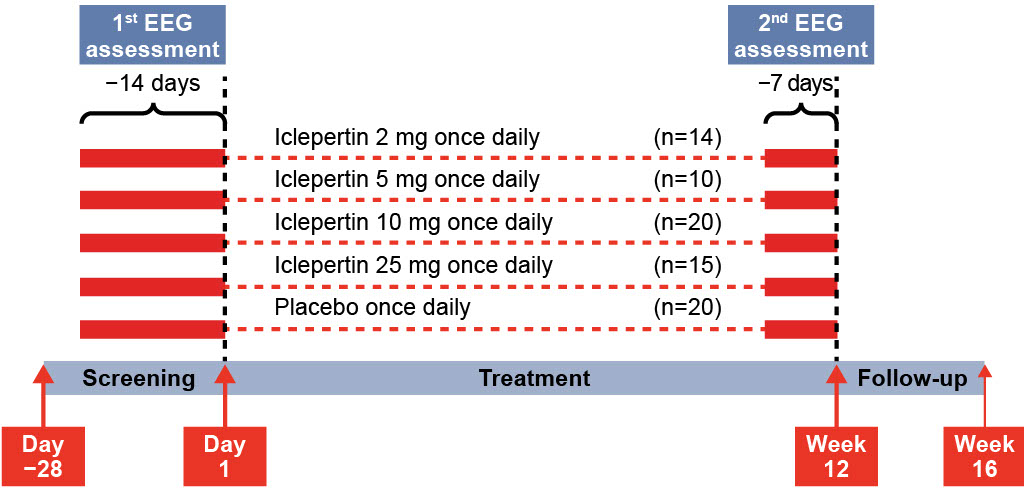

Supplement: Supplementary file 2 — Supplementary Figure 1 [file 41398_2022_2096_MOESM2_ESM.jpg]

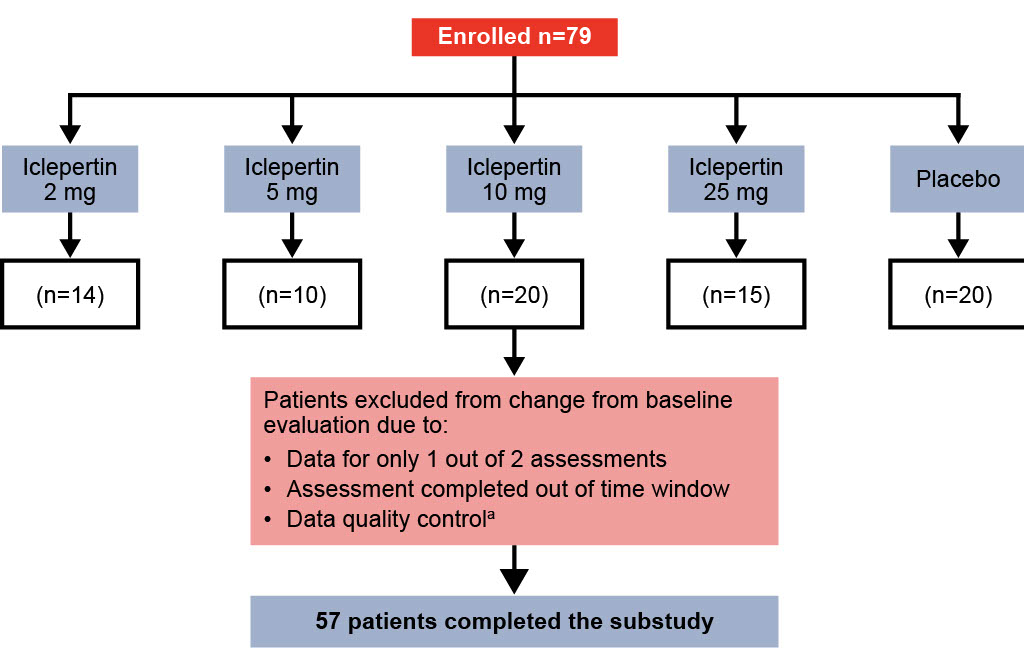

Supplement: Supplementary file 3 — Supplementary Figure 2 [file 41398_2022_2096_MOESM3_ESM.jpg]

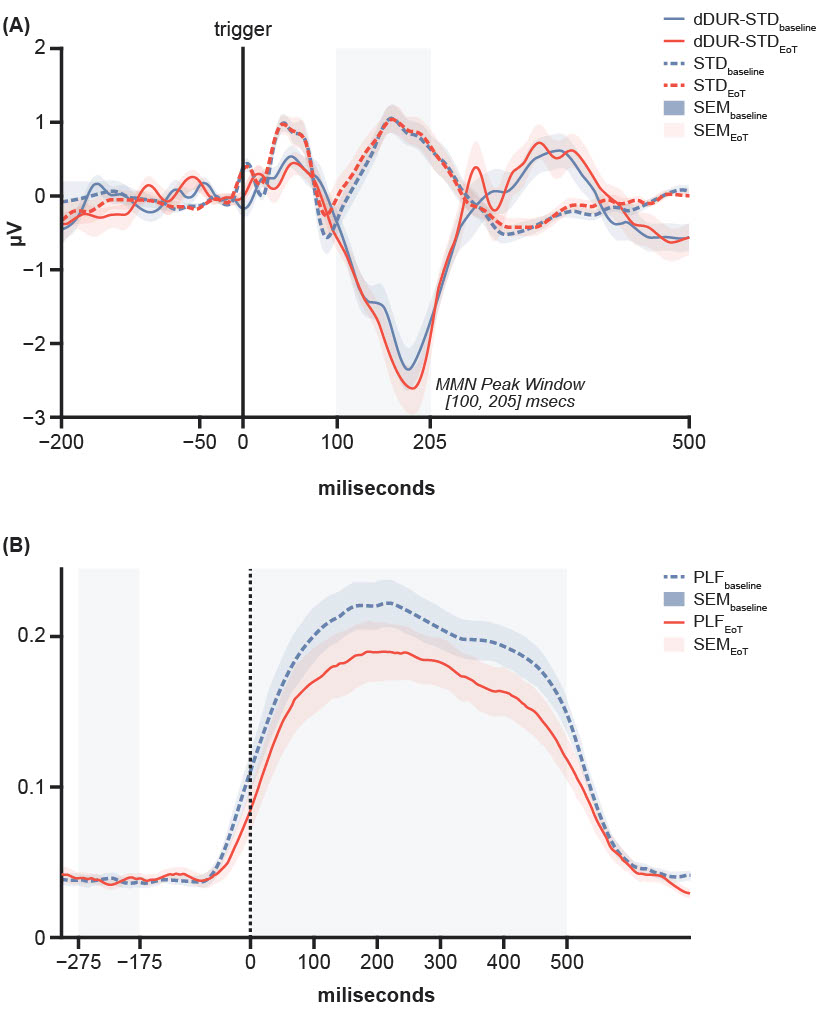

Supplement: Supplementary file 4 — Supplementary Figure 3 [file 41398_2022_2096_MOESM4_ESM.jpg]

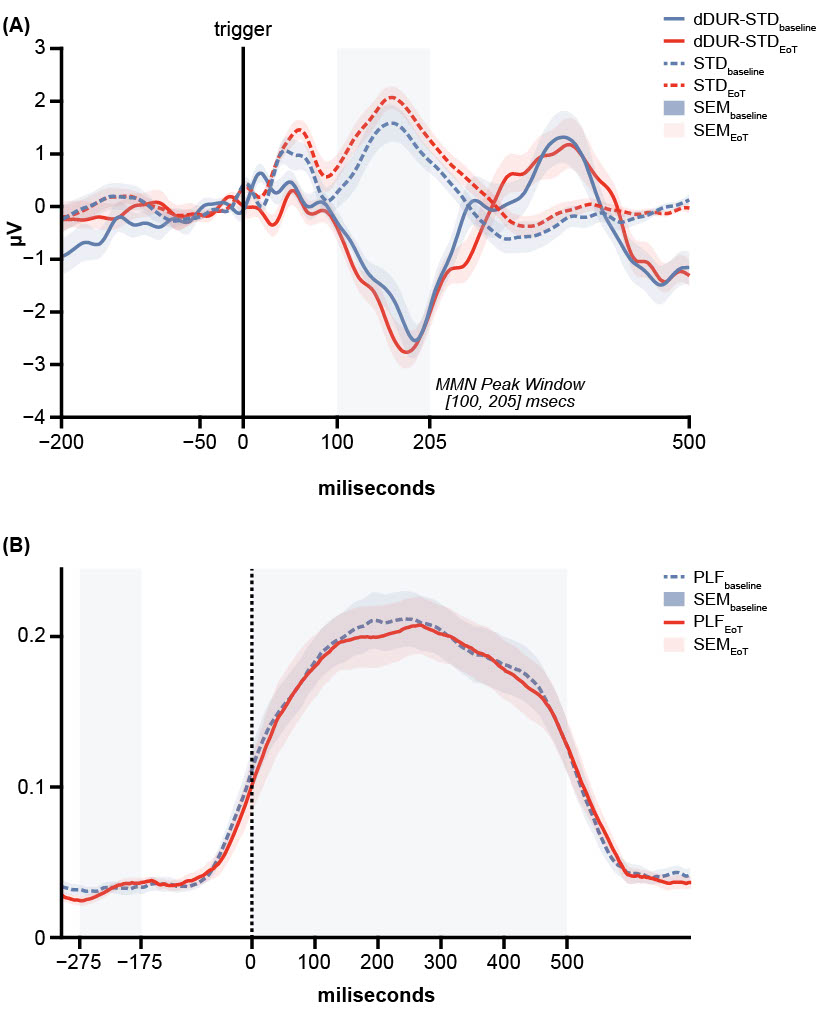

Supplement: Supplementary file 5 — Supplementary Figure 4 [file 41398_2022_2096_MOESM5_ESM.jpg]
